# Supplementary material for: Efficacy and safety of dofetilide and sotalol in patients with hypertrophic cardiomyopathy
Source: Commun Med (Lond). 2023 Jul 19;3:99. doi: 10.1038/s43856-023-00315-8 (PMC10356938; doi:10.1038/s43856-023-00315-8)
Supplement: Supplementary file 1 — Supplementary Information [file 43856_2023_315_MOESM1_ESM.pdf]

Supplemental Table 1 Total daily doses of DOF and STL according to arrhythmia and proportion of events at that dose

	**Arrhythmia Recurrence** Number at dose with recurrence / total number at dose (%)	**Discontinuation** Number at dose when discontinued/ total number at dose (%)
**DOF – AF (Total Daily Dose in mcg) ***		
250	0/1 (0.0%)	0/1 (0.0%)
500	5/7 (71.4%)	1/7 (14.3%)
750	1/2 (50%)	0/2 (0.0%)
1000	3/9 (33.3%)	7/10 (70.0%)
**STL – AF (Total Daily Dose in mg) ****		
80	3 / 6 (50.0%)	1 / 6 (16.7%)
160	9 / 19 (47.4%)	5 / 19 (26.3%)
240	7 / 13 (53.8%)	6 / 14 (42.9%)
320	4 / 10 (40%)	3 / 11 (27.2%)
480	0 / 1 (0.0%)	0 / 1 (0.0%)
**STL – VA (Total Daily Dose in mg)**		
80	0 /1 (0.0%)	0 /1 (0.0%)
160	2 /4 (50.0%)	2 / 4 (50.0%)
240	5 /7 (71.4%)	3 /7 (42.9%)
320	1 /3 (33.3%)	3 /3 (100%)
400	1/1 (100%)	0/1 (0.0%)
480	0/1 (0.0%)	0/1 (0.0%)

*1 patient on 500mcg stopped for inefficacy after 5 days and thus censored from the Arrhythmia Recurrence column
**1 patient on 160mg stopped after LVH finding on echo and thus censored from the Arrhythmia Recurrence column. Another patient censored from both columns due to lack of details including medication start date, dose, and follow-up details

Supplemental Table 2 Multivariate Cox proportional hazard models of predictors for drug discontinuation.

	HR	95% CI	P-value
Hypertension	0.08	0.01-0.78	0.030
Obstructive Sleep Apnea	0.18	0.05-0.73	0.017
LVEF	0.89	0.83-0.95	<0.001
IVSd	0.18	0.04-0.79	0.023

Wald test p=0.02

Supplemental Table 3 Univariate cox regression assessing predictors of arrhythmia recurrence of both drugs by time of chart review for all patients (N=144)

	HR	95% CI	P- value
Age	0.987	0.975, 0.999	0.034
Gender	1.064	0.718, 1.575	0.758
Race	0.495	0.295, 0.829	0.008
Hypertension	1.007	0.688, 1.473	0.972
Diabetes	1.827	1.165, 2.864	0.009
Obstructive Sleep Apnea	1.066	0.732, 1.552	0.739
Tobacco Smoking	0.985	0.568, 1.707	0.956
Chronic Kidney Disease	1.096	0.531, 2.261	0.804
Congestive Heart Failure	0.867	0.563, 1.337	0.519
NYHA Class	0.939	0.698, 1.263	0.678
Paroxysmal AF	0.779	0.457, 1.328	0.359
Persistent AF	0.781	0.523, 1.165	0.225
Atrial Flutter	1.039	0.622, 1.735	0.884
History of sustained VT	1.344	0.901, 2.006	0.148
LVEF	0.995	0.976, 1.013	0.569
LA diameter PLAX	0.910	0.832, 0.996	0.040
LAVI	0.994	0.978, 1.010	0.468
IVSd	0.738	0.500, 1.091	0.128
SAM	1.141	0.710, 1.833	0.586
Obstructive Gradient	1.148	0.753, 1.750	0.521
Resting LVOT gradient	1.001	0.995, 1.006	0.862
Valsalva LVOT gradient	1.001	0.996, 1.006	0.753
Exercise LVOT gradient	1.012	1.000, 1.023	0.053

Supplemental Table 4 Univariate cox regression assessing predictors of discontinuation of both drugs by time of chart review for all patients (N=144)

	HR	95% CI	P- value
Age	1.009	0.994, 1.024	0.243
Gender	1.061	0.685, 1.642	0.792
Race	0.687	0.366, 1.291	0.244
Hypertension	1.167	0.769, 1.771	0.469
Diabetes	1.317	0.802, 2.163	0.277
Obstructive Sleep Apnea	0.831	0.548, 1.259	0.383
Tobacco Smoking	1.478	0.815, 2.682	0.199
Chronic Kidney Disease	1.786	0.855, 3.730	0.123
Congestive Heart Failure	1.730	1.108, 2.702	0.016
NYHA Class	1.469	1.084, 1.990	0.013
Paroxysmal AF	0.790	0.446, 1.399	0.419
Persistent AF	1.091	0.693, 1.719	0.707
Atrial Flutter	1.292	0.737, 2.265	0.371
History of sustained VT	0.974	0.617, 1.539	0.911
LVEF	0.959	0.939, 0.980	<0.001
LA diameter PLAX	0.902	0.810, 1.003	0.057
LAVI	1.000	0.980, 1.021	0.987
IVSd	0.918	0.645, 1.306	0.634
SAM	1.371	0.817, 2.301	0.233
Obstructive Gradient	1.426	0.895, 2.271	0.135
Resting LVOT gradient	1.000	0.993, 1.008	0.922
Valsalva LVOT gradient	1.001	0.995, 1.007	0.772
Exercise LVOT gradient	1.062	1.029, 1.096	<0.001

Supplemental Table 5 Univariate Cox analysis for predictors of arrhythmia recurrence by time of chart review for all patients on STL (N=59)

	HR	95% CI	P- value
Age	0.987	0.968, 1.006	0.170
Gender	1.356	0.732, 2.512	0.333
Race	0.485	0.230, 1.025	0.058
Hypertension	1.098	0.602, 2.002	0.760
Diabetes	1.650	0.838, 3.251	0.148
Obstructive Sleep Apnea	0.933	0.515, 1.691	0.820
Tobacco Smoking	0.758	0.316, 1.821	0.536
Chronic Kidney Disease	1.020	0.314, 3.313	0.973
Congestive Heart Failure	0.744	0.376, 1.473	0.396
NYHA Class	0.849	0.526, 1.371	0.503
Paroxysmal AF	0.685	0.288, 1.631	0.393
Persistent AF	0.780	0.422,1.443	0.428
Atrial Flutter	0.982	0.452, 2.133	0.963
History of sustained VT	1.552	0.835, 2.883	0.165
LVEF	0.991	0.962, 1.021	0.561
LA diameter PLAX	0.910	0.789, 1.048	0.190
LAVI	0.986	0.960, 1.013	0.300
IVSd	0.803	0.454, 1.418	0.449
SAM	1.056	0.501, 2.228	0.885
Obstructive Gradient	1.088	0.551, 2.149	0.807
Resting LVOT gradient	0.999	0.990, 1.008	0.856
Valsalva LVOT gradient	1.000	0.994, 1.007	0.896
Exercise LVOT gradient	1.042	1.008, 1.078	0.016

Supplemental Table 6 Univariate Cox analysis for predictors of discontinuation by time of chart review for all patients on STL (N=59)

	HR	95% CI	P- value
Age	1.013	0.989, 1.038	0.293
Gender	1.056	0.522, 2.138	0.879
Race	0.622	0.247, 1.567	0.314
Hypertension	1.220	0.619, 2.404	0.565
Diabetes	1.487	0.701, 3.153	0.301
Obstructive Sleep Apnea	0.868	0.442, 1.705	0.681
Tobacco Smoking	1.341	0.512, 3.509	0.550
Chronic Kidney Disease	3.112	0.889, 10.900	0.076
Congestive Heart Failure	1.700	0.828, 3.490	0.148
NYHA Class	1.475	0.901, 2.417	0.123
Paroxysmal AF	0.971	0.391, 2.413	0.949
Persistent AF	0.876	0.431, 1.779	0.714
Atrial Flutter	1.171	0.480, 2.858	0.729
History of sustained VT	1.108	0.534, 2.300	0.783
LVEF	0.963	0.930, 0.998	0.041
LA diameter PLAX	0.916	0.796, 1.054	0.218
LAVI	1.006	0.974, 1.039	0.722
IVSd	0.912	0.522, 1.594	0.748
SAM	1.334	0.589, 3.018	0.490
Obstructive Gradient	1.484	0.698, 3.154	0.305
Resting LVOT gradient	1.000	0.988, 1.012	0.979
Valsalva LVOT gradient	1.001	0.992, 1.009	0.880
Exercise LVOT gradient	1.059	1.007, 1.112	0.025

Supplemental Table 7 Univariate Cox analysis for predictors of AF recurrence by time of chart review for patients on STL (n=49)

	HR	96% CI	P- value
Age	0.990	0.970, 1.010	0.315
Gender	1.188	0.617, 2.287	0.606
Race	0.302	0.113, 0.804	0.017
Hypertension	1.046	0.543, 2.015	0.892
Diabetes	1.418	0.641, 3.141	0.389
Obstructive Sleep Apnea	0.981	0.504, 1.911	0.956
Tobacco Smoking	0.870	0.358, 2.113	0.758
Chronic Kidney Disease	1.443	0.340, 6.116	0.619
Congestive Heart Failure	1.121	0.548, 2.291	0.754
NYHA Class	1.059	0.658, 1.704	0.813
Paroxysmal AF	0.880	0.364, 2.129	0.777
Persistent AF	0.935	0.424, 2.062	0.867
Atrial Flutter	1.381	0.623, 3.060	0.427
History of sustained VT	1.331	0.615, 2.882	0.468
LVEF	0.968	0.932, 1.005	0.093
LA diameter PLAX	0.929	0.831, 1.038	0.192
LAVI	0.994	0.961, 1.029	0.735
IVSd	0.923	0.508, 1.676	0.791
SAM	1.254	0.579, 2.713	0.566
Obstructive Gradient	1.077	0.508, 2.287	0.846
Resting LVOT gradient	1.000	0.992, 1.009	0.913
Valsalva LVOT gradient	1.001	0.995, 1.008	0.698
Exercise LVOT gradient	1.036	1.003. 1.069	0.031

Supplemental Table 8 Univariate Cox analysis for predictors of discontinuation by time of chart review for AF patients on STL

	HR	95% CI	P- value
Age	1.012	0.987, 1.039	0.355
Gender	1.031	0.488, 2.178	0.937
Race	0.733	0.214, 2.509	0.621
Hypertension	0.967	0.464, 2.013	0.928
Diabetes	1.301	0.548, 3.089	0.552
Obstructive Sleep Apnea	0.669	0.320, 1.396	0.284
Tobacco Smoking	1.383	0.517, 3.697	0.519
Chronic Kidney Disease	1.318	0.172, 10.130	0.791
Congestive Heart Failure	1.627	0.751, 3.524	0.217
NYHA Class	1.480	0.872, 2.513	0.146
Paroxysmal AF	0.982	0.389, 2.478	0.969
Persistent AF	0.923	0.387, 2.205	0.857
Atrial Flutter	0.998	0.375, 2.261	0.996
History of sustained VT	0.887	0.348, 2.261	0.801
LVEF	0.967	0.929, 1.007	0.107
LA diameter PLAX	0.907	0.773, 1.064	0.232
LAVI	0.997	0.955, 1.041	0.905
IVSd	0.901	0.505, 1.609	0.725
SAM	1.600	0.682, 3.752	0.280
Obstructive Gradient	1.778	0.807, 3.915	0.153
Resting LVOT gradient	0.999	0.988, 1.011	0.925
Valsalva LVOT gradient	1.000	0.991, 1.009	0.991
Exercise LVOT gradient	1.091	0.999, 1.191	0.052

Supplemental Table 9 Univariate Cox analysis for predictors of AF recurrence by time of chart review for patients on DOF (N=19)

	HR	95% CI	P- value
Age	0.996	0.961, 1.032	0.815
Gender	0.339	0.103, 1.118	0.075
Hypertension	0.723	0.256, 2.043	0.540
Diabetes	2.175	0.459, 10.300	0.328
Obstructive Sleep Apnea	1.526	0.529, 4.403	0.434
Tobacco Smoking	1.945	0.420, 9.007	0.395
Chronic Kidney Disease	0.930	0.206, 4.192	0.925
Congestive Heart Failure	2.001	0.677, 5.918	0.210
NYHA Class	1.859	0.913, 3.784	0.087
Paroxysmal AF	1.395	0.176, 11.090	0.753
Persistent AF	0.717	0.090, 5.696	0.753
Atrial Flutter	0.821	0.182, 3.709	0.798
History of sustained VT	3.068	0.358, 26.310	0.307
LVEF	1.009	0.924, 1.102	0.843
LA diameter PLAX	1.005	0.636, 1.589	0.983
LAVI	1.026	0.976, 1.078	0.313
IVSd	0.825	0.174, 3.914	0.809
SAM	0.389	0.086, 1.768	0.222
Obstructive Gradient	1.036	0.326, 3.296	0.952
Resting LVOT gradient	1.000	0.969, 1.033	0.979
Valsalva LVOT gradient	1.023	0.986, 1.061	0.233

Supplemental Table 10 Univariate Cox analysis for predictors of discontinuation by time of chart review for AF patients on DOF (N=19)

	HR	95% CI	P- value
Age	0.977	0.932, 1.024	0.322
Gender	0.747	0.223, 2.503	0.637
Hypertension	0.621	0.205, 1.875	0.398
Diabetes	0.718	0.091, 5.627	0.752
Obstructive Sleep Apnea	1.039	0.346, 3.124	0.946
Tobacco Smoking	2.809	0.564, 14.000	0.207
Congestive Heart Failure	1.717	0.511, 5.766	0.382
NYHA Class	1.196	0.566, 2.531	0.639
Atrial Flutter	4.770	1.041, 21.870	0.044
History of sustained VT	1.565	0.194, 12.610	0.674
LVEF	0.990	0.916, 1.069	0.792
LA diameter PLAX	0.840	0.392, 1.797	0.653
LAVI	0.897	0.778, 1.034	0.134
IVSd	2.901	0.726, 11.590	0.132
SAM	4.342	1.029, 18.320	0.046
Obstructive Gradient	1.407	0.374, 5.301	0.613
Resting LVOT gradient	1.066	0.999, 1.138	0.052
Valsalva LVOT gradient	1.046	0.983, 1.113	0.156

Supplemental Table 11 Univariate Cox analysis for predictors of VT recurrence by time of chart review for patients on STL (N=17)

	HR	95% CI	P- value
Age	0.969	0.927, 1.014	0.174
Gender	0.786	0.207, 2.989	0.724
Race	0.519	0.145, 1.863	0.314
Hypertension	0.863	0.272, 2.739	0.803
Diabetes	4.670	1.151, 18.940	0.031
Obstructive Sleep Apnea	1.537	0.463, 5.106	0.483
Tobacco Smoking	2.664	0.310, 22.860	0.372
Chronic Kidney Disease	1.075	0.135, 8.558	0.946
Congestive Heart Failure	0.193	0.024, 1.560	0.123
NYHA Class	0.222	0.032, 1.565	0.131
Paroxysmal AF	0.995	0.262, 3.779	0.995
Persistent AF	0.127	0.016, 1.013	0.051
History of sustained VT	3.992	0.505, 31.570	0.190
LVEF	1.033	0.976, 1.094	0.262
LA diameter PLAX	0.807	0.416, 1.566	0.526
LAVI	0.992	0.949, 1.036	0.706
IVSd	0.261	0.048, 1.404	0.118
SAM	1.951	0.234, 16.270	0.537
Obstructive Gradient	1.025	0.264, 3.986	0.971
Resting LVOT gradient	1.050	0.961, 1.146	0.280
Valsalva LVOT gradient	0.986	0.943, 1.030	0.516
Exercise LVOT gradient	0.986	0.942, 1.032	0.536

Supplemental Table 12 Univariate Cox analysis for predictors of discontinuation by time of chart review for VT patients on STL (n=17)

	HR	95% CI	P- value
Age	1.018	0.969, 1.069	0.482
Gender	0.873	0.218, 3.491	0.847
Race	0.818	0.200, 3.348	0.780
Hypertension	2.020	0.520, 7.844	0.310
Diabetes	1.436	0.358, 5.759	0.609
Obstructive Sleep Apnea	1.700	0.509, 5.686	0.389
Tobacco Smoking	7.485	0.678, 82.610	0.100
Chronic Kidney Disease	9.887	1.360, 71.890	0.024
Congestive Heart Failure	3.013	0.841, 10.790	0.090
NYHA Class	3.728	1.177, 11.810	0.025
Paroxysmal AF	0.649	0.132, 3.186	0.595
Persistent AF	1.953	0.556, 6.870	0.297
Atrial Flutter	15.490	0.969, 247.8	0.053
History of sustained VT	0.839	0.174, 4.053	0.827
LVEF	0.912	0.851, 0.977	0.009
LA diameter PLAX	0.936	0.408, 2.144	0.875
LAVI	1.017	0.972, 1.063	0.471
IVSd	1.455	0.306, 6.914	0.637
Obstructive Gradient	1.324	0.330, 5.317	0.692
Resting LVOT gradient	1.030	0.921, 1.152	0.600
Valsalva LVOT gradient	1.015	0.973, 1.059	0.497
Exercise LVOT gradient	1.023	0.978, 1.070	0.320
