# Supplementary material for: Efficacy and safety of dofetilide and sotalol in patients with hypertrophic cardiomyopathy
Source: Commun Med (Lond). 2023 Jul 19;3:99. doi: 10.1038/s43856-023-00315-8 (PMC10356938; doi:10.1038/s43856-023-00315-8)
Supplement: Supplementary file 2 — Reporting Summary [file 43856_2023_315_MOESM2_ESM.pdf]

nature portfolio

Corresponding author(s): Christopher Chen, Babak Nazer

Last updated by author(s): 5/25/2023

Reporting Summary

Nature Portfolio wishes to improve the reproducibility of the work that we publish. This form provides structure for consistency and transparency in reporting. For further information on Nature Portfolio policies, see our Editorial Policies and the Editorial Policy Checklist.

Statistics

For all statistical analyses, confirm that the following items are present in the figure legend, table legend, main text, or Methods section.

n/a	Confirmed	
☐	☒	The exact sample size (n) for each experimental group/condition, given as a discrete number and unit of measurement
☒	☐	A statement on whether measurements were taken from distinct samples or whether the same sample was measured repeatedly
☐	☒	The statistical test(s) used AND whether they are one- or two-sided<br>*Only common tests should be described solely by name; describe more complex techniques in the Methods section.*
☐	☒	A description of all covariates tested
☐	☒	A description of any assumptions or corrections, such as tests of normality and adjustment for multiple comparisons
☒	☐	A full description of the statistical parameters including central tendency (e.g. means) or other basic estimates (e.g. regression coefficient) AND variation (e.g. standard deviation) or associated estimates of uncertainty (e.g. confidence intervals)
☐	☒	For null hypothesis testing, the test statistic (e.g. F, t, r) with confidence intervals, effect sizes, degrees of freedom and P value noted<br>*Give P values as exact values whenever suitable.*
☒	☐	For Bayesian analysis, information on the choice of priors and Markov chain Monte Carlo settings
☒	☐	For hierarchical and complex designs, identification of the appropriate level for tests and full reporting of outcomes
☒	☐	Estimates of effect sizes (e.g. Cohen's d, Pearson's r), indicating how they were calculated

Our web collection on statistics for biologists contains articles on many of the points above.

Software and code

Policy information about availability of computer code

Data collection	Epic Electronic Medical Record (specifically, Epic's Slicer Dicer function) and Microsoft Excel
Data analysis	R Statistical Software

For manuscripts utilizing custom algorithms or software that are central to the research but not yet described in published literature, software must be made available to editors and reviewers. We strongly encourage code deposition in a community repository (e.g. GitHub). See the Nature Portfolio guidelines for submitting code & software for further information.

Data

Policy information about availability of data

All manuscripts must include a data availability statement. This statement should provide the following information, where applicable:

- Accession codes, unique identifiers, or web links for publicly available datasets
- A description of any restrictions on data availability
- For clinical datasets or third party data, please ensure that the statement adheres to our policy

Data sets were generated as a .csv file from extracted patient information from the electronic medical record system of our hospital institution. They are currently stored within a secure Microsoft OneDrive storage system owned by our academic hospital institution, and are available from the corresponding authors upon request. Source data behind Figure 1 specifically can be viewed in Supplementary Data 1.

Human research participants

Policy information about studies involving human research participants and Sex and Gender in Research.

Reporting on sex and gender	This was not a project focused on sex/gender.
Population characteristics	Patients had diagnoses or histories of Hypertrophic cardiomyopathy with or without obstruction, atrial fibrillation and/or ventricular arrhythmias and were taking dofetilide or sotalol.
Recruitment	Recruitment was not performed. This was a retrospective analysis.
Ethics oversight	IRB approved at the study site's institution

Note that full information on the approval of the study protocol must also be provided in the manuscript.

Field-specific reporting

Please select the one below that is the best fit for your research. If you are not sure, read the appropriate sections before making your selection.

☒ Life sciences ☐ Behavioural & social sciences ☐ Ecological, evolutionary & environmental sciences

For a reference copy of the document with all sections, see nature.com/documents/nr-reporting-summary-flat.pdf

Life sciences study design

All studies must disclose on these points even when the disclosure is negative.

Sample size	Sample size based on what data existed and if it met specified inclusion criteria
Data exclusions	Original patient list was curated and then filtered if it did not meet specified exclusion criteria
Replication	N/A - this is a retrospective analysis
Randomization	Not randomized, this is a retrospective analysis
Blinding	Not blinded, this is a retrospective analysis

Reporting for specific materials, systems and methods

We require information from authors about some types of materials, experimental systems and methods used in many studies. Here, indicate whether each material, system or method listed is relevant to your study. If you are not sure if a list item applies to your research, read the appropriate section before selecting a response.

Materials & experimental systems

n/a	Involved in the study	
☒	☐	Antibodies
☒	☐	Eukaryotic cell lines
☒	☐	Palaeontology and archaeology
☒	☐	Animals and other organisms
☒	☐	Clinical data
☒	☐	Dual use research of concern

Methods

n/a	Involved in the study	
☒	☐	ChIP-seq
☒	☐	Flow cytometry
☒	☐	MRI-based neuroimaging
