# Supplementary material for: Efficacy and safety of dofetilide and sotalol in patients with hypertrophic cardiomyopathy
Source: Commun Med (Lond). 2023 Jul 19;3:99. doi: 10.1038/s43856-023-00315-8 (PMC10356938; doi:10.1038/s43856-023-00315-8)
Supplement: Supplementary file 4 — Description of Additional Supplementary Files [file 43856_2023_315_MOESM4_ESM.pdf]

Description of Additional Supplementary Files

File name: Supplementary Data 1
Description: Source data behind Figure 1.
